# Modelling the implementation of narrow versus broader spectrum antibiotics in the empiric treatment of *E. coli* bacteraemia

**DOI:** 10.1038/s41598-024-66193-9

**Published:** 2024-07-23

**Authors:** Mark P. Khurana, Jacob Curran-Sebastian, Samir Bhatt, Gwenan M. Knight

**Affiliations:** 1 Section of Epidemiology, Department of Public Health, University of Copenhagen, Øster Farimagsgade 5, 1352 Copenhagen, Denmark; 2 MRC Centre for Global Infectious Disease Analysis, School of Public Health, Imperial College London, London, SW7 2AZ UK; 3 Department of Infectious Disease Epidemiology, Faculty of Epidemiology and Population Health, AMR Centre, Centre for Mathematical Modeling of Infectious Diseases, London School of Hygiene and Tropical Medicine (LSHTM), London, UK

**Keywords:** Bacteria, Clinical microbiology, Policy and public health in microbiology

## Abstract

The implementation of new antimicrobial resistance stewardship programs is crucial in optimizing antibiotic use. However, prescription choices can be difficult during empiric therapy; clinicians must balance the survival benefits of broader spectrum antibiotics with associated increases in resistance. The aim of this study was to evaluate the overall feasibility of switching to narrow spectrum antibiotics during the empiric treatment of *E. coli* bacteraemia by quantifying changes in resistance rates, antibiotic usage, and mortality using a deterministic state-transition model. Three unique model scenarios (A, B, and C), each representing a progressively broader spectrum empiric treatment regimen, were used to compare outcomes at 5 years. We show that the empiric use of the narrowest spectrum (first-line) antibiotics can lead to reductions in resistance to second-line antibiotics and the use of third-line antibiotics, but they also lead to increases in resistance to first-line therapy and higher mortality. Crucially, we find that shortening the duration of empiric and overall treatment, as well as reducing the baseline mortality rate, are important for increasing the feasibility of switching to narrow spectrum antibiotics in the empiric treatment of *E. coli* bacteraemia. We provide a flexible model design to investigate optimal treatment approaches for other bacterial infections.

## Introduction

Antimicrobial resistance (AMR) is a growing health concern, with the situation exacerbated by a growing number of multi-resistant organisms and a corresponding dearth of new antibiotics^1,2^. New antibiotic stewardship approaches are therefore needed to reduce the rate of resistance^3^. A principal antibiotic stewardship strategy is more selectively choosing which antibiotics to use in practice. For example, a key tenet of the United Kingdom’s 5-year national action plan for tackling AMR (2019–2024) is the optimization of prescribing practices^4^; ideally, antibiotics are chosen based on local epidemiological patterns and current resistance rates^5^. Additionally, from a stewardship perspective, narrow spectrum antibiotics (referred to as first-line antibiotics in this article) are often preferred to broader spectrum ones (second- and third-line antibiotics) because they exert more specific selection pressure for resistance; over time, this should reduce the number of multi-resistant organisms^6^.

However, the choice of which antibiotic to prescribe is not simple. Firstly, the causative organism and its resistance phenotype are often unknown. This is particularly true early in the infection when treatment is given in the absence of complete clinical information (empiric therapy), and the choice of antibiotic is based on experience and/or local knowledge^7^. Secondly, clinicians are faced with a number of competing interests^8^; on the one hand, the clinician has a duty of care and is responsible for the survival of the patient, particularly when infections are severe^9^, and can therefore be nudged towards prescribing broader spectrum antibiotics to ensure that the treatment covers the causative microorganism^10^. Since inappropriate empiric therapy is a risk factor for mortality, many clinicians are justifiably hesitant in switching to narrow spectrum antibiotics to avoid increasing the patient’s likelihood of death in cases where the spectrum is not effective against the as-yet, unknown causative organism^11,12^. On the other hand, prescribing broader spectrum antibiotics could contribute to growing resistance due to the wider coverage of these antibiotics—we are more concerned about resistance to these broadly effective drugs^2,13,14^.

From a policy perspective, being able to implement policies and guidelines that minimize the future negative effects of AMR while guaranteeing sufficient treatment for current patients is critical. For example, the World Health Organization (WHO) launched the AWaRe (Access, Watch, Restrict) classification system in 2017 to help decide which antibiotics should be preferred based on their resistance profiles and microbiological activity^15^. Mathematical modeling can also be a useful tool in helping decide how and when certain antibiotics should be used. The number of AMR modeling studies has been steadily increasing, highlighting the potential of these studies to better inform policy^16,17^. In the context of empiric therapy, modeling can remove some of the burden placed on individual clinicians attempting to predict how their actions will influence both patient survival and changes in resistance, allowing us to systematically quantify which strategies are worth pursuing^16,18^.

A key infection syndrome which requires optimized empiric treatment is bacteraemia. *E. coli* is the most common cause of bacteraemia in high-income countries^19^, and the most common source of community-acquired bacteraemia^20^. Treating *E. coli* bacteraemia is a classic case study of the clinical dilemma faced by prescribing doctors. On the one hand, mortality rates for patients with *E. coli* bacteraemia are high. In one systematic review, the case fatality rate (CFR) was found to be 12.4% (95% CI 10.7–14.3%)^19^. In other studies, 7-day mortality rates vary between 6.7% and 8.5%^21–26^. As such, there is a strong incentive towards picking broader spectrum empiric treatment out of precaution. On the other hand, *E. coli* resistance rates are increasing, with multi-drug strains more challenging to treat and more likely to result in death^25,27,28^. This would incentivize using narrower spectrum antibiotics during empiric therapy to reduce the rate at which multi-drug resistance develops in *E. coli* isolates.

This study develops a new state-transition model to capture the stages in empiric therapy to explore the impact of different empiric treatment strategies on resistance rates, antibiotic usage, and clinical outcomes over time. By quantifying each of these elements, the aim is to evaluate the overall merits of switching to narrow spectrum antibiotics during empiric therapy, and the extent to which it is a feasible stewardship solution in the context of *E. coli* bacteraemia.

## Results

**Figure 1:**
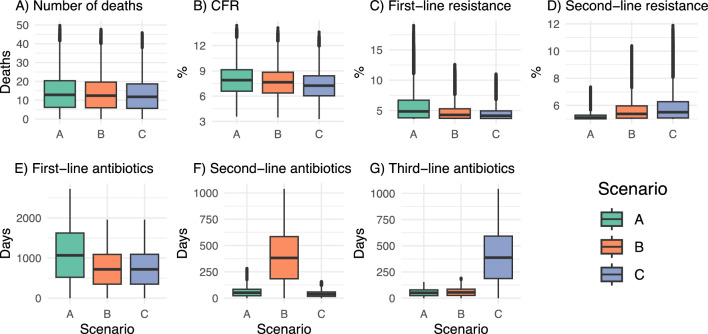
Box plots with the distribution of baseline outcome measures at five years across all runs (n = 1000), stratified by scenario. The baseline scenario was seven days of treatment, of which two days were empiric therapy. *CFR* time-updated case fatality rate.

For each of the three scenarios (A-C), baseline results were calculated for the main outcome measures of interest at five years (Table 1, Fig. 1). We consistently found that mortality was highest in Scenario A (first-line empiric therapy) compared to both B (second-line empiric therapy) and C (third-line empiric therapy). The CFR was 0.69% (95%CI [0.30, 1.08]) and 0.41% (95%CI [0.20, 0.62]) higher for Scenarios A and B, respectively, compared to Scenario C at five years. Across all runs, first-line resistance was 2.29% (95%CI [0.08, 4.50]) and 1.86% (95%CI [0.40, 3.32]) higher for Scenario A, on average, compared to C and B respectively. Second-line resistance, however, was consistently higher for scenarios B (0.90% [0.46, 1.34]) and C (1.32% [0.21, 2.43]) compared to A. Interestingly, use of third-line antibiotics was similar for Scenarios A and B, despite the increases in second-line resistance due to use of second-line antibiotics during empiric therapy (Fig. 1G). The results therefore highlight that despite increases in resistance to second-line therapy and the substantial increases in the volume of second- and third-line treatment provided, significant mortality benefits remained when using second- and third-line empiric therapy. Notably, however, there was significant variation in the estimates (Fig. 1) across all outcome measures, highlighting both the uncertainty of parameter values and the sensitivity of the model to these parameters.

**Table 1:** Baseline mean scenario-specific value of outcome measures and mean outcome differences between corresponding (i.e., paired) runs in each scenario at 5 years across all runs (n = 1000), including standard deviations (given in parentheses, SD).

Outcome measure (standard deviation)	Scenario A	Scenario B	Scenario C	Difference, A − B	Difference, B − C	Difference, A − C
Number of deaths	28.3 (6.7)	27.3 (6.4)	25.9 (6.2)	1.03 (0.36)	1.45 (0.38)	2.48 (0.73)
CFR, %	7.95 (1.87)	7.66 (1.80)	7.25 (1.73)	0.29 (0.10)	0.41 (0.11)	0.69 (0.20)
% Resistance to first-line therapy	7.68 (3.26)	5.82 (1.81)	5.39 (1.49)	1.86 (1.50)	0.44 (0.36)	2.29 (1.86)
% Resistance to second-line therapy	5.44 (4.16)	6.34 (1.13)	6.76 (1.47)	−**0.90 (0.73)**	−**0.42 (0.34)**	−**1.32 (1.07)**
# Days with first-line treatment	2178.8 (143.4)	1475.3 (156.1)	1475.6 (156.1)	703.5 (83.9)	−**0.14 (0.13)**	703.3 (83.9)
# Days with second-line treatment	132.8 (37.8)	797.3 (80.3)	86.5 (18.7)	−**664.5 (82.5)**	710.8 (83.8)	46.3 (23.5)
# Days with third-line treatment	108.5 (10.6)	121.3 (15.8)	805.5 (81.9)	−**12.8 (7.5)**	−**684.2 (80.5)**	−**696.9 (81.9)**

The baseline scenario was seven days of treatment, of which two days were empiric therapy. Bolded cells indicate a negative value.

**Figure 2:**
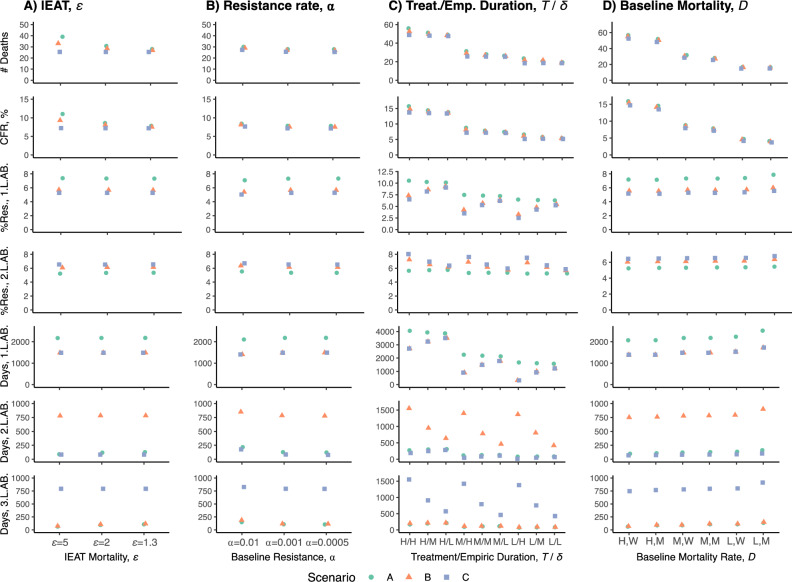
Graphs with outcome measure results at five years for different combinations of parameter values: (**A**) inappropriate empiric antibiotic therapy (IEAT), ε, (**B**) resistance transmission rate, ⍺, (**C**) treatment and empiric duration, *T* and 1/*δ*. Treatment durations (*T*) were split into 5 (L), 7 (M), and 14 (H) days of treatment. For empiric therapy (*δ*), values were 4 (H), 2 (M), and 1 (L) day of empiric treatment. (**D**) Baseline mortality rate, *D;* Differences in mortality between those infected with pan-susceptible, 1st and second-line resistant microorganisms were split into marginal (M) (*D*_*2*_ = 1.5*D, D_*3*_ = 2*D) and wide (W) (*D*_*2*_ = 2*D, D_*3*_ = 4*D). Results are further stratified by Scenario A–C, denoted by shape and color for clarity. All other parameters kept at baseline values as in Table 3. *L.AB*. line antibiotic.

To explore the role of individual parameter values on the results, we conducted sensitivity analyses with a range of values for the variable parameters. For inappropriate empiric antibiotic therapy (IEAT), we found that reducing the survival proportion (ε) during inappropriate therapy resulted in a significantly higher number of deaths and higher CFRs for Scenario A, relatively smaller increases for Scenario B and no changes for Scenario C (Fig. 2A), which is expected given the broader spectrum of antibiotics in B and C. However, changes in the survival proportion (ε) resulted in negligible changes in resistance rates and the number of treatment days (Fig. 2A). When adjusting breakthrough resistance rates (⍺), we found that mortality marginally decreased with lower ⍺ values, although relative differences remained similar between scenarios A-C (Fig. 2B). Higher breakthrough resistance rates values resulted in negligible changes in population-level resistance to first- and second-line therapy but resulted in fewer days with first-line treatment and marginal increases in the number of days with second- and third-line therapy, although the magnitude of this effect was small (Fig. 2B).

While the model suggests that mortality increases with increasing treatment duration (Fig. 2C), this is expected given the structure of the model (since the mortality parameter is given per day, extending the number of days in which patients can experience death). The increases in mortality therefore reflect the combined contribution of the model structure and increases in resistance rates due to prolonged therapy. We also found that mortality increases as the duration of empiric therapy increases (Fig. 2C), a finding consistent across all three scenarios. Resistance to first- and second-line therapy, as well as the number of days of treatment, followed similar trends. Predictably, the CFR increased as the baseline death rate and mortality differences between infections with pan-susceptible, first-line and second-line resistant organisms increased (Fig. 2D). Resistance to first- and second-line therapy increased with lower mortality rates and with more marginal mortality differences between resistance phenotypes, with a similar trend emerging for the number of days with first- and second-line antibiotics (Fig. 2D).

**Figure 3:**
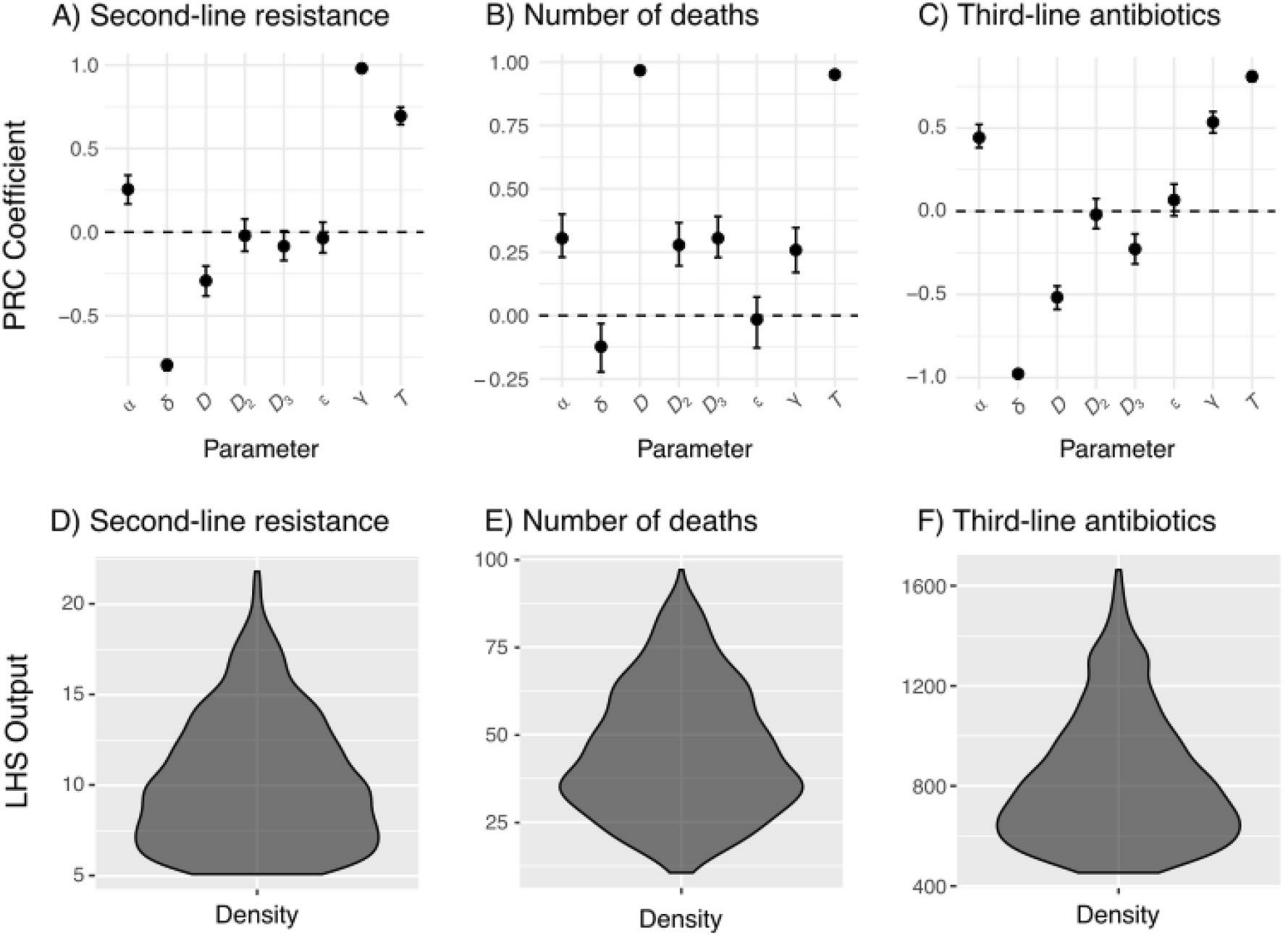
Top row: partial rank correlation (PRC) coefficients (with 95% CIs) for second-line resistance (**A**), number of deaths (**B**), and third-line antibiotic (**C**) use at 5 years, using scenario A as a baseline. Bottom row: violin plots with density distributions for corresponding Latin hypercube sampling (LHS) output values (**D–F**). Note that empiric therapy duration (in days) is 1/*δ*.

Multivariate sensitivity analysis revealed that for second-line resistance, there was strong evidence that resistance transmission (*γ*), treatment duration (*T*) and empiric therapy duration (1/*δ*) were correlated with second-line resistance (Figs. 3A and 5D). Additionally, increases in baseline mortality rates (*D*) were correlated with second-line resistance, while higher breakthrough resistance rates (*⍺*) were also strongly correlated with second-line resistance. Interestingly, only inappropriate empiric therapy (ε) was not correlated with mortality (Figs. 3B and 5E), suggesting that other factors are driving the associated increases in mortality seen in the earlier sensitivity analyses. Lastly, for third-line antibiotic use, we found that treatment duration and empiric therapy duration (1/*δ*) were most strongly correlated with third-line antibiotic use (Figs. 3C and 5F). Baseline mortality rates (*D* and *D*_*3*_) and resistance transmission (*γ*) were also correlated with third-line antibiotic use, although CIs were wider and the correlations weaker. Overall, a broad range of output values was evident, highlighting the uncertainty inherent in outcome measures and the extensive potential impacts stemming from diverse parameter values (Fig. 3D–F).

## Discussion

A significant clinical dilemma facing clinicians is whether to empirically prescribe narrow- or broad-spectrum antibiotics, balancing clinical success with reductions in the use of broad-spectrum antibiotics^8–10^. In this study, we find that empiric prescribing with narrower spectrum antibiotics (scenarios A and to a lesser extent B) can lead to reductions in resistance over 5 years to second-line broader treatment, as well as large reductions in the use of third-line broad spectrum antibiotics in the context of *E. coli* bacteremia. However, use of narrower spectrum antibiotics as first-line empiric therapy also led to increases in resistance to first-line therapy as well as increases in mortality. Despite the reduction in resistance to second-line therapy and broad-spectrum antibiotic use, the significant relative reductions in mortality justify why many clinicians choose to prescribe broader spectrum antibiotics. Given the conservative mortality and inappropriate therapy parameter estimates used, the true mortality differences between scenarios are likely to be greater than in the baseline scenario, particularly in settings with fewer healthcare resources. As such, the results mirror the uncertainty encountered in clinical settings when determining the appropriate antibiotics for empiric therapy, especially in the context of *E. coli* bacteremia, where no regimen stands out as comparatively superior in terms of both clinical success and long-term resistance.

A key finding is the effect of empiric therapy duration; we found that shorter empiric treatment durations resulted in large decreases in mortality for scenario A, while those for B and C were less impacted. The main explanation for this effect is that patients experience less treatment time with inappropriate antibiotic therapy, thus improving their chances of survival. Additionally, the number of treatment days (for first-, second-, and third-line therapy) and resistance rates (to first- and second-line therapy) was marginally reduced with shorter empiric durations. From a policy perspective, shortening empiric therapy durations is a key intervention in increasing the feasibility of switching to narrow spectrum antibiotics, particularly in places with high baseline mortality rates. Crucially, this is also achievable in high-resource settings. New diagnostic methods and point-of-care testing can improve the speed in which organisms are identified and antimicrobial susceptibility testing can be completed^29–31^. From a logistics perspective, shorter turnaround times from sample-taking to results, achieved through novel diagnostic methods, overnight testing, automation, and process improvements have been demonstrated to be effective^32–35^. We also note that the implementation of narrow spectrum antibiotics as empiric therapy is much more feasible in high-resource settings due to baseline mortality rates being lower. To aid the adoption of narrower-spectrum antibiotics as first-line therapy, another solution is to risk-stratify. Since certain demographics are known to have higher baseline mortality rates^36^, stratifying them into low- and high-risk patients could result in a split strategy whereby some patients receive first-line therapy while those at higher risk receive second- or third-line treatment, a scenario we did not explore here.

Direct comparison with the extant modeling literature is challenging because few articles have addressed optimal empiric therapy from the perspective of narrow versus broad spectrum antibiotics, despite this being a key AMR policy question. In fact, it constitutes a central facet of the WHO’s AWaRe (Access, Watch, Restrict) initiative. As part of the WHO’s 13th General Programme of Work 2019–2023, the WHO has set a country-level objective of achieving a minimum of 60% of total antibiotic consumption being from Access group antibiotics, which are mostly narrow-spectrum, thus shifting antibiotic use from broad- to narrow-spectrum^37,38^. Thus far, mathematical modeling of empiric therapy has mostly been concerned with general principles, such as whether monotherapy, combination therapy, mixing (i.e., randomly assigning patients to different antibiotics), or cycling (i.e., scheduled changes in first-line antibiotics) results in the most optimal resistance outcomes^39–42^. Given the lack of empirical data to help guide model calibration, it is not surprising that previous research has focused on general principles rather than focusing on specific clinical situations^17^. For example, Gjini et al. found that if treatment is initiated sufficiently early, then short and strong (i.e., high dosage) treatments are beneficial, whereas mild and long regimens are preferable if treatment starts late^43^. In Brazil, a study based on local susceptibility patterns suggested that a combination of at least three antibiotics was necessary to achieve adequate empirical therapy coverage, as monotherapy and even dual therapy options were found to be insufficient, particularly in intensive care units and wards^44^. Another modeling study, using an individual-based model of hospital outbreaks, found that shorter treatment durations were associated with fewer antibiotic resistance epidemics in hospitals^45^. These findings correspond to those in this study, where treatment duration was found to be an important driver of resistance to both first- and second-line therapy.

The approach in this study has several limitations. Firstly, the model is deterministic and does not capture the full random variation of biological processes, including the stochasticity of organisms developing resistance. We also assume that *E. coli* is the causative organism of the bacteraemia; in reality, *E. coli* competes with many other microorganisms that could also cause the infection. Additionally, the model assumes that patients receive treatment for fixed durations and then either die or recover, as well as assuming that patient characteristics are homogenous. Previous studies have also demonstrated that there are mortality differences by age and sex with regards to *E. coli* bacteraemia^24,26,46,47^, and that the distribution of resistance differs by age and sex, with a higher percentage of resistance (to ciprofloxacin in this case) among men and among those aged 15- to 44-years old^48^.

Well-defined parameter values are also scarce. Data regarding the rate at which patients develop resistance during treatment is lacking, specifically regarding resistance rates for specific combinations of antibiotics, such as how frequently a patient with ESBL-producing *E. coli* bacteria develops resistance when treated with meropenem compared with cephalosporins. This scarcity becomes even more pronounced when looking for rates when being treated with combination therapy. Our *γ* parameter, denoting the increase in population-level resistance per day of treatment, is also prone to uncertainty^49^. Firstly, it encompasses a wide range of phenomena: continued colonization of patients, transfer of mobile genetic elements (MGE), and other indirect AMR transmission mechanisms^50,51^. Inevitably, this composite parameter is likely to be uncertain, since interactions between patients, environmental factors, and the stochastic nature of mutations all interact^51,52^. Additionally, the degree to which the use of specific antibiotic combinations leads to increases in resistance in the population (i.e., how specific antibiotics mediate *γ*) was based on correlation data, which is likely to be context-specific^53,54^. Overall, many parameter values were uncertain or lacking in the literature, which has been noted previously by others^17^. Collecting data to update these values would not only improve the accuracy of the model used in this study but would also be a boon for future AMR modeling work.

## Conclusion

Our results suggest that the use of narrow spectrum antibiotics can be a viable empiric treatment option for *E. coli* bacteraemia, although other modifiable factors related to treatment (e.g., shortening the duration of empiric therapy and reducing baseline mortality rates) are crucial in improving the feasibility of this switch. Additionally, shortening treatment durations can have significant positive effects on reducing increases in resistance to both first- and second-line therapies. However, in settings with higher mortality rates and where empiric therapy is prolonged, switching to narrow spectrum antibiotics results in mortality increases that render the strategy unfeasible, despite reductions in second-line resistance and third-line antibiotic use. The flexible model design used in this study is also well-placed to explore whether these treatment dynamics persist for other bacterial infections and strains.

## Methods

### Model design

**Figure 4:**
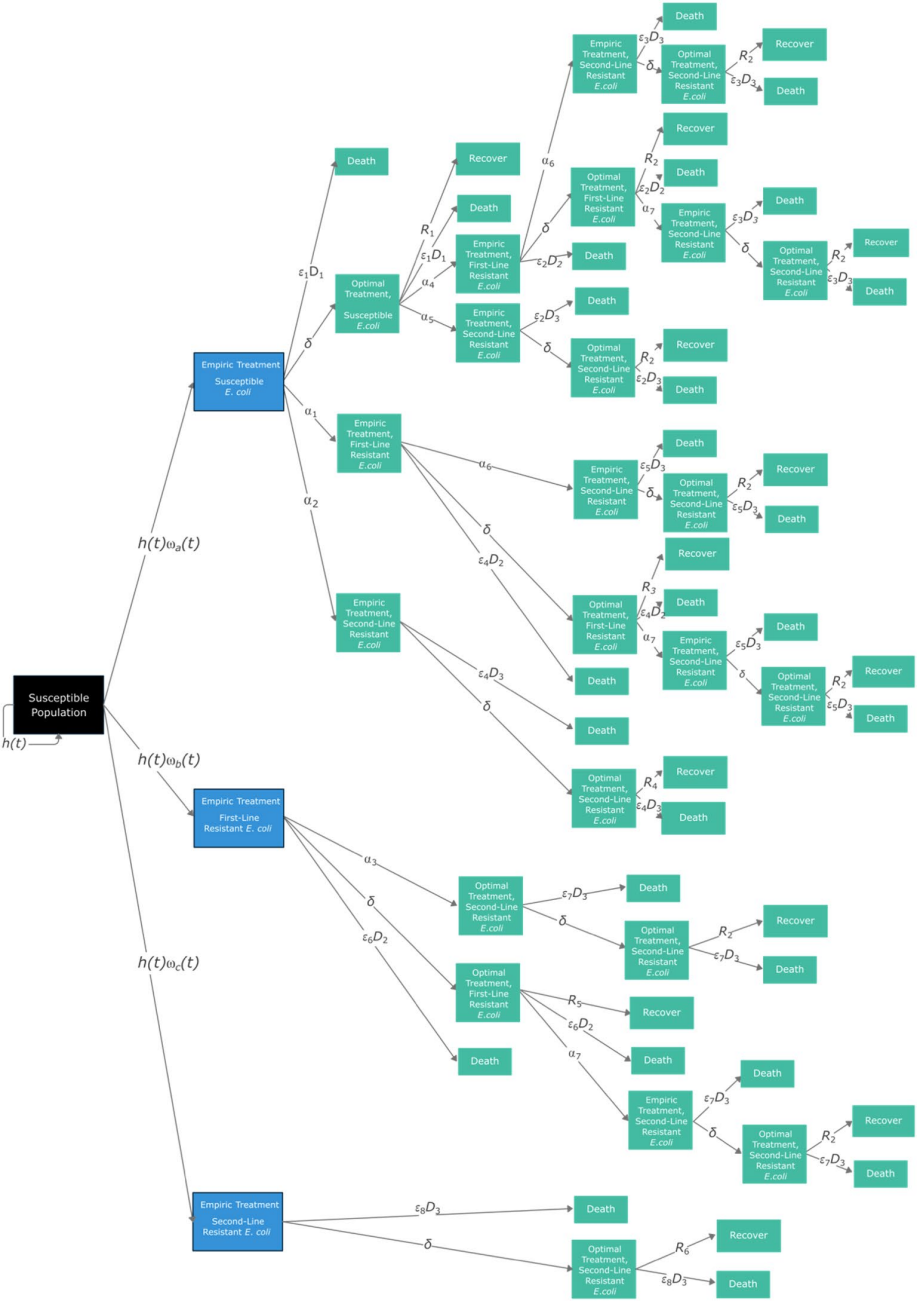
*E. coli* bacteraemia state-transition model. Black denotes the susceptible cohort; blue denotes which compartments are directly affected by scenarios A–C; green boxes are consistent across models (expanded in Fig. 5). Parameter notation is as in Table 3.

We use a deterministic state-transition model to fit a variety of patient pathways for *E. coli* bacteraemia (model structure shown in Fig. 4). Movement between compartments was modeled using ordinary differential equations (ODEs) and was coded in the statistical software *R* (version 4.2.2), including the packages *Tidyverse* and *deSolve*^55–57^. *R* code in Supplementary S1 and Zenodo: 10.5281/zenodo.10354268. The susceptible population (*N*_*s*_ $\in {Z}^{+})$ is a theoretical population cohort, with *E. coli* bacteraemia hospitalization rates (*h *$\in {R}^{+}$*, λ* $\in {R}^{+}$) and resistance phenotype distributions (*⍵* $\in [\text{0,1}]$) based on values from the United Kingdom as a baseline case study. The time horizon was five years. Each day, a proportion of the susceptible cohort (100,000 *N*_*s*_) are assumed to be hospitalized with bacteraemia (*h*(*t*)), with the proportion with resistance varying over time—mediated by the gamma (*γ*) rate—and then started on empiric treatment (either Scenario A, B, or C).

**Figure 5:**
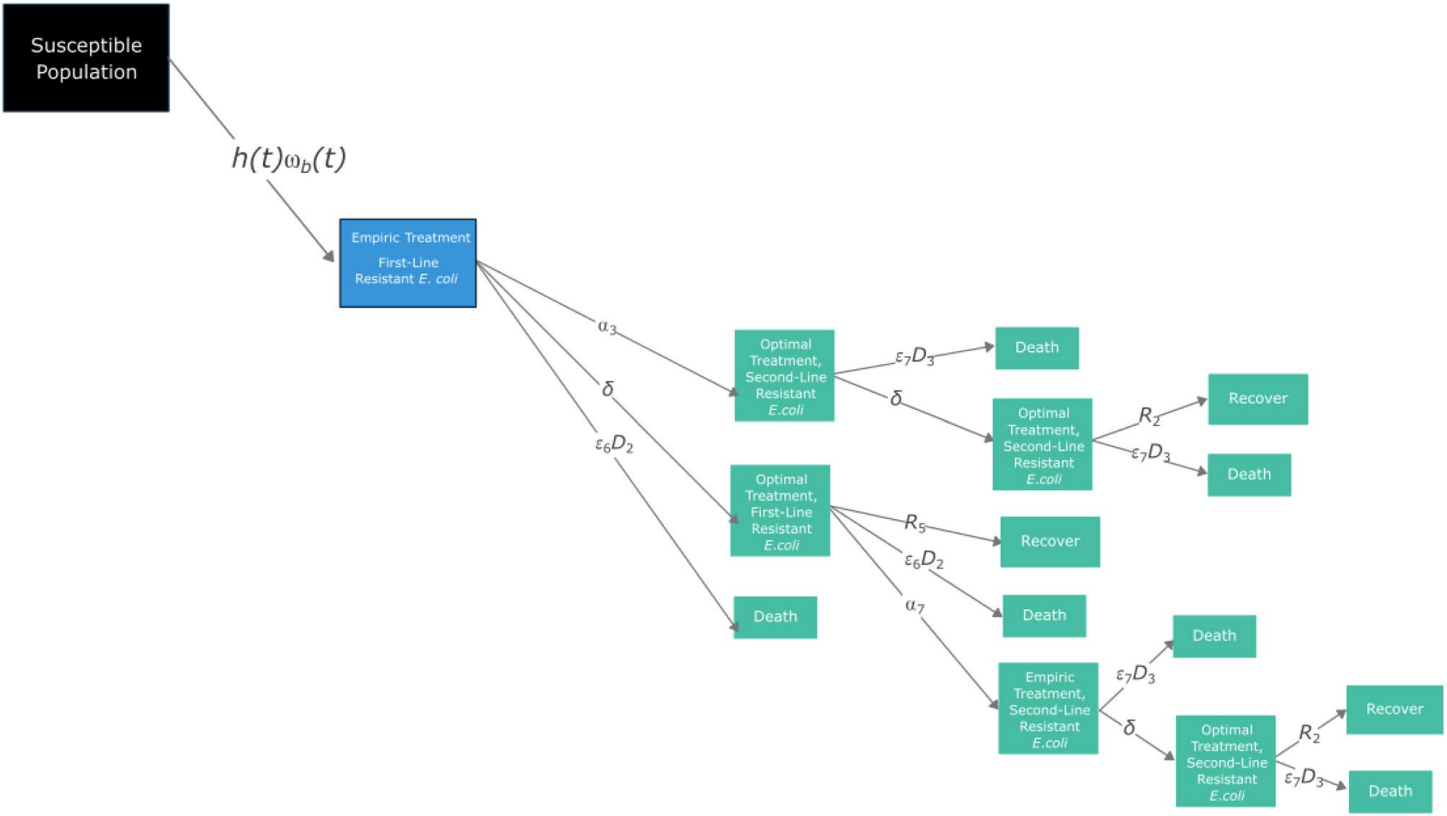
Partial component of Fig. 4 for illustrative purposes. Black denotes the susceptible population; blue denotes the compartment directly affected by scenarios A–C; green boxes are consistent across scenarios A–C. Parameter notation is as in Fig. 4.

To explain the structure of the model, a subset of Fig. 4 can be seen in Fig. 5. The potential pathways for patients with pan-susceptible (i.e., susceptible to first-, second- and third-line antibiotics), first-line resistant (i.e., susceptible to second- and third-line antibiotics) or second-line resistant (i.e., susceptible to third-line antibiotics) *E. coli* after starting each scenario (A, B, and C) is the same (Fig. 5). Patients move from the susceptible population to a hospitalized empiric treatment group; in Fig. 5, the rate for this transition is *h(t)⍵*_b_(*t*). Note that parameter subscripts are numbered to denote the specific conditions governing the transition between two compartments, which are formally described in Table 3. During this empiric treatment stage (of length [1/*δ*]), the causative organism and resistance phenotype is unknown. The treatment the patient receives in the model is thus dependent on the scenario being modeled (A, B, and C, described later). Inappropriate treatment is accounted for by the parameter ε_*,*_ which leads to an increased mortality rate that depends on the treatment given. Acquisition of resistance is governed by the parameter ⍺. During the empiric treatment phase, patients can die (rate: ε_6_*D*_2_), develop resistance to the next line therapy (rate: ⍺_3_) or move to the optimal treatment compartment (rate: *δ*). The rate at which optimal treatment is initiated, *δ*, is dependent on the organism and phenotype being identified, as well as appropriate treatment being initiated.

During the optimal treatment period for first-line resistant *E. coli*, the patient can recover (rate: *R*_5_), die (rate: ε_6_*D*_2_), or develop resistance to second-line treatment (rate: ⍺_7_). The rate of recovery is dependent on whether the empiric therapy given covered the microorganism and its resistance phenotype. For example, if the microorganism is susceptible to the empiric therapy, the duration of optimal treatment is equal to the fixed total treatment duration (*T*) minus the already experienced empiric treatment duration. However, if the microorganism is not susceptible to the empiric treatment therapy, the optimal treatment period is equal to the fixed total treatment duration, *T*. If the causative microorganism develops resistance during treatment, either during the empiric treatment phase or optimal treatment phase, then a new empiric treatment phase starts since the resistance phenotype being responded to is outdated. This captures the clinical realities of treating infections with outdated information, requiring new samples to be taken before optimal treatment is restored.

### Outcome measures

The main outcome measures fall within three broad categories: mortality metrics, resistance levels and antibiotic usage. For both baseline and sensitivity analyses, outcomes are measured at 5 years. The main outcome measures are: (1) total number of deaths; (2) time-updated CFR (%) [number of deaths/(number of deaths + number recovered)]; (3) percent (%) resistance to first-line therapy; (4) percent (%) resistance to second-line therapy; (5) cumulative number of days of first-, second-, and third-line treatment.

### Model scenarios, parameterization, and assumptions

Three distinct model scenarios (A, B, and C) were compared, each representing a different initial empiric therapy choice (Table 2). Antibiotic guidance for bacteraemia and sepsis from NICE in the UK is not standardized, with clinicians deferring to local antimicrobial guidance^58^. While the WHO provides guidance on the empiric treatment of sepsis through the *WHO AWaRe *(*Access, Watch, Reserve*)* antibiotic book*, guidance is not specific to isolated cases of bacteraemia^59^. Initial empiric therapy is also often dependent on patient risk factors, including previous antimicrobial treatment, severity of illness, and the presumed source of the infection^20,60,61^. To standardize treatment regimens for *E. coli* bacteraemia, the regimens in this study are based on a combination of NICE guidelines, guidance from the Infectious Disease Society of America and expert opinion from the UK used in a previous study^58,60,62^. Broadly speaking, first-line therapy is a beta-lactam antibiotic combined with an aminoglycoside (Table 2)^63^. Second-line therapy is often a second- or third-generation cephalosporin with an aminoglycoside, and third-line therapy is commonly a carbapenem^63^. These treatment regimens will also reflect the different empiric therapy scenarios tested in the study, outlined in more detail in the next section. The scenarios represent the empiric therapy choice at the start of the infection (blue compartments in Fig. 4) allowing a comparison of how narrow versus broader spectrum empiric antibiotic treatment influences resistance rates, antibiotic usage, and clinical outcomes.

**Table 2:** *E. coli* bacteraemia treatment regimens and model scenarios.

Empiric therapy	Antibiotic 1	Antibiotic 2	Spectrum
First-line (scenario A)	Co-amoxiclav (beta-lactam and beta-lactamase inhibitor)	Gentamicin (aminoglycoside)	Narrow
Second-line (scenario B)	Cefuroxime (second-generation cephalosporin)	Gentamicin (aminoglycoside)	Broader
Third-line (scenario C)	Meropenem (carbapenem)		Broad

Parameters were sourced where possible from the literature (Table 3). The initial prevalence of resistance to each therapy (*⍵*) and several of the patient pathway values (*h, λ*) were based on data from the UK^64^. The model assumes that clinicians act homogeneously with regards to their clinical practice, such that other treatment aspects (e.g., oxygen, intravenous fluids, etc.) are consistent. Additionally, we assume that blood samples for bacterial culture are taken immediately, that blood cultures have 100% sensitivity and specificity, and that patient characteristics are uniform (e.g., homogenous age, comorbidities, and sex). It is also assumed that patients are correctly diagnosed as having bacteraemia, that the infection is not influenced by other secondary diagnoses (i.e., no interaction between diagnoses), and that patients either fully recover or die after their treatment is complete. As such, refractory infections (e.g., treatment failure or prolonged infections) are not accounted for. It is also assumed that the background population (i.e., those who are not yet hospitalized) is closed, where births and deaths among are omitted, such that the population size (*N*_*s*_*)* is kept constant (i.e., the population is replenished at the same rate that people are hospitalized).

**Table 3:** List of parameters for all scenarios (A-C) and unique parameter values for each scenario (A, B, C), including baseline values, range for sensitivity analyses and references.

Symbol	Parameter	Baseline value	Range for sensitivity analyses	Notes and references
Common parameter values for all scenarios (A–C)				
* N*_*s*_	Susceptible population	100,000		Fixed, theoretical cohort. Replenished at the rate of *h*(*t*) (i.e., rate of hospitalization), such that it is kept constant
**δ**	Rate (per day) of switch to optimal treatment	0.5 per day (i.e., mean 2 days empiric therapy duration)	0.25–1 i.e., one to four days of empiric treatment	^65^, explored during sensitivity analysis (low, medium, and high *δ* values)
* ⍵*(*t*)	Percent of *E. coli* bacteria with resistance phenotype a, b, and c	⍵_a_ = 0.914 ⍵_b_ = 0.036 ⍵_c_ = 0.050		^66^, explored during sensitivity analysis. Updates over time based on *γ*. Phenotypes a-c correspond to pan-susceptible, first-and second-line resistant respectively
* γ*	Increase in population-level resistance per day of treatment with a given antibiotic; denoted resistance transmission rate	1.8 × 10^–5^	1 × 10^–6^ and 1 × 10^–4^	^67^, explored during sensitivity analysis. Alters the value of *⍵* over time
* h*(*t*)	Population rate (per day) of *E. coli* bacteremia hospitalization	1.88 × 10^–6^		Most recent estimate from the UK; 68.5 per 100,000 per year^64^ On each day, h*N_s_*(*1* + *λ*) individuals are hospitalized. *h(t)* is therefore mediated by *λ*
* λ*	Daily rate of increase in *E. coli* bacteremia hospitalizations	4.66 × 10^–5^ (1.7% per year/365 days)		UK estimates for increase in incidence between 2021 and 2023^64^; follows a linear trend (Supplementary S3); converted to daily rate
*** D***	Rate of death per day during treatment	*D* = 0.01 (baseline) *D*_1_ = *D* *D*_2_ = D*1.5 *D*_3_ = D*2	*D* = 0.005 to 0.02 *D*_2_ and *D*_3_ between 1.5 and 4 × higher than D	^21,22,24,26,68,69^, explored during sensitivity analysis *D* = 0.01 (baseline) *D*_1_ = *D* (infection with pan-susceptible organism) *D*_2_ = D*1.5 (infection with first-line resistant organism) *D*_3_ = D*2 (infection with second-line resistant organism)
* R*	Rate of recovery	*R*_1_ = 1/(*T*-(1/δ))		Dependent on total treatment duration
		*R*_2_ = 1/*T*		
**⍺**	Rate of (breakthrough) resistance during therapy	Baseline ⍺ = 0.001, or 0.1% per day ⍺_4_ = ⍺ ⍺_5_ = ⍺/5 ⍺_6_ = ⍺ ⍺_7_ = ⍺*1.5	⍺: 0.0005–0.01	See Supplementary S4 and S5 for derivation ⍺_4_ = rate of resistance during optimal therapy from a pan-susceptible to first-line resistant strain ⍺_5_ = rate of resistance during optimal therapy from a pan-susceptible to second-line resistant strain ⍺_6_ = rate of resistance during empiric therapy from a first-line resistant to second-line resistant strain ⍺_7_ = rate of resistance during optimal therapy from a first-line resistant to second-line resistant strain ⍺_1–3_ appear in subsequent rows
*** T***	Total treatment duration	7 days	5, 7, 14 days	Fixed in baseline, explored during sensitivity analysis ^70,71^
Parameters for scenario A (co-amoxiclav/gentamicin)				
**ε**	Reduced survival due to inappropriate empiric antibiotic therapy (IEAT) (i.e., increase in mortality associated with each day of IEAT)	ε = 1.3^(1/δ)/2^ (baseline)	1.1^(1/δ)/2^ to 5^(1/δ)/2^	^11,72^, explored during sensitivity analyses
		ε_1,2_ = ε^o^ (appropriate therapy)		
		ε_4,6,8_ = ε (one inappropriate therapy period)		
		ε_3,5,7_ = ε^2^ (two inappropriate therapy periods)		
* R*	Rate of Recovery (per day)	R_3-6_ = 1/*T*		
**⍺**	Rate of (breakthrough) resistance during therapy (per day)	⍺_1_ = ⍺ ⍺_2_ = ⍺/5 ⍺_3_ = ⍺		See Supplementary S5 for derivation
Parameters for scenario B (cefuroxime/gentamicin)				
**ε**	Reduced survival due to inappropriate empiric therapy (IEAT)	ε = 1.3^(1/δ)/2^ (baseline)	1.3^(1/δ)/2^ to 5^(1/δ)/2^	^11,72^, explored during sensitivity analyses
		ε_1,2,4,6_ = ε^o^ (appropriate therapy)		
		ε_3,5,7,8_ = ε (one inappropriate therapy period)		
* R*	Rate of recovery	R_3_ = 1/(*T* − (1/δ)) R_4_ = 1/*T* R_5_ = 1/(*T* − (1/δ)) R_6_ = 1/*T*		
**⍺**	Rate of (breakthrough) resistance during therapy	⍺_1_ = ⍺*1.375 ⍺_2_ = ⍺*1.125 ⍺_3_ = ⍺*1.5		See Supplementary S5 for derivation
Parameters for scenario C (meropenem)				
**ε**	Reduced survival due to inappropriate empiric therapy (IEAT)	ε = 1.3^(1/δ)/2^ (baseline)	1.1^(1/δ)/2^ to 5^(1/δ)/2^	^11,72^, explored during sensitivity analyses
		ε_1-8_ = ε^o^ (appropriate therapy)		
* R*	Rate of recovery	R_3-6_ = 1/(*T* − (1/*δ*))		
**⍺**	Rate of (breakthrough) resistance during therapy	⍺_1_ = ⍺*1.375 ⍺_2_ = ⍺*1.125 ⍺_3_ = ⍺*1.5		See Supplementary S5 for derivation

Further details given in Supplementary S2. Variable parameter symbols are highlighted in bold.

### Baseline and sensitivity analyses

For the baseline analysis, to explore uncertainty, a normal distribution was fit to several highly uncertain or setting-dependent parameters (i.e., treatment duration [*T*], empiric therapy duration [1/*δ*], breakthrough resistance rates [⍺], the effect of inappropriate therapy [ε], population-level effects of antibiotic use on resistance levels [*γ*], and baseline mortality rates [*D*]), which we denote variable parameters. The mean for each variable parameter was set to the sourced baseline parameter value (Table 3), with standard deviations (SD) set such that the 95% CI of the distribution was within the ranges used for the sensitivity analyses, described later (Supplementary S1). We ran 1000 iterations of the model for each scenario, sampling independently from the distributions of the variable parameters for each run while keeping non-variable parameters constant. To make results comparable between scenarios, the same set of parameter values was used for scenarios A-C in each corresponding run. Mean and SD values for scenario-specific values of outcome measures and outcome differences between corresponding (i.e., paired) runs in each scenario at five years were calculated. SDs for the differences between scenarios were calculated based on the distribution of paired differences between corresponding runs.

For sensitivity analyses, we split variable parameter values into low, medium, and high values (Table 3). Treatment durations (*T*) were split into 5 (low), 7 (medium), and 14 (high) days of treatment. For the empiric therapy duration (1/*δ*), high, medium, and low values were 4, 2, and 1 day of empiric treatment. Baseline breakthrough resistance rates (⍺) were split into 0.0005, 0.001, and 0.01 (i.e., 0.05%, 0.1% and 1% per day). Baseline inappropriate empiric antibiotic therapy (ε) values were split into 1.3, 2, and 5 respectively. Mortality rates (*D*) were stratified into high (0.02), medium (0.01), and low (0.005) values (i.e., 0.5%, 1% and 2% mortality per day). Differences in mortality between those infected with pan-susceptible, first- and second-line resistant microorganisms were also split into marginal (*D*_2_ = 1.5*D, *D*_3_ = 2*D) and wide (*D*_2_ = 2*D, *D*_3_ = 4*D). Multivariate sensitivity analyses were conducted using Latin Hypercube Sampling (1000 samples). Partial rank correlation coefficients with 95% CIs were calculated for each parameter.

### Supplementary Information

Supplementary Information.

## Data Availability

The datasets generated and/or analysed during the current study are available on Zenodo at 10.5281/zenodo.10354268.
