# Supplementary material for: Modelling the implementation of narrow versus broader spectrum antibiotics in the empiric treatment of E. coli bacteraemia
Source: Sci Rep. 2024 Jul 23;14:16986. doi: 10.1038/s41598-024-66193-9 (PMC11266692; doi:10.1038/s41598-024-66193-9)
Supplement: Supplementary file 1 — Supplementary Information. [file 41598_2024_66193_MOESM1_ESM.pdf]

SUPPLEMENTARY MATERIAL

Running head: Modelling *E. coli* bacteraemia treatment

Modelling the implementation of narrow versus broader spectrum antibiotics in the empiric treatment of *E. coli* bacteraemia

Mark P. Khurana[1], Jacob Curran-Sebastian[1], Samir Bhatt[1,2], Gwenan M Knight[3]

[1]*Section of Epidemiology, Department of Public Health, University of Copenhagen, 1352 Copenhagen, Denmark*

[2]*MRC Centre for Global Infectious Disease Analysis, School of Public Health, Imperial College London, SW7 2AZ, London, UK*

[3]*Department of Infectious Disease Epidemiology, Faculty of Epidemiology and Population Health, London School of Hygiene and Tropical Medicine (LSHTM), London, UK*

Supplementary S1

R Code and Figure for Model, Illustrations and Sensitivity Analyses

Zenodo link: [10.5281/zenodo.10354268](https://doi.org/10.5281/zenodo.10354268)

Tree diagram with compartment names used in the R code:

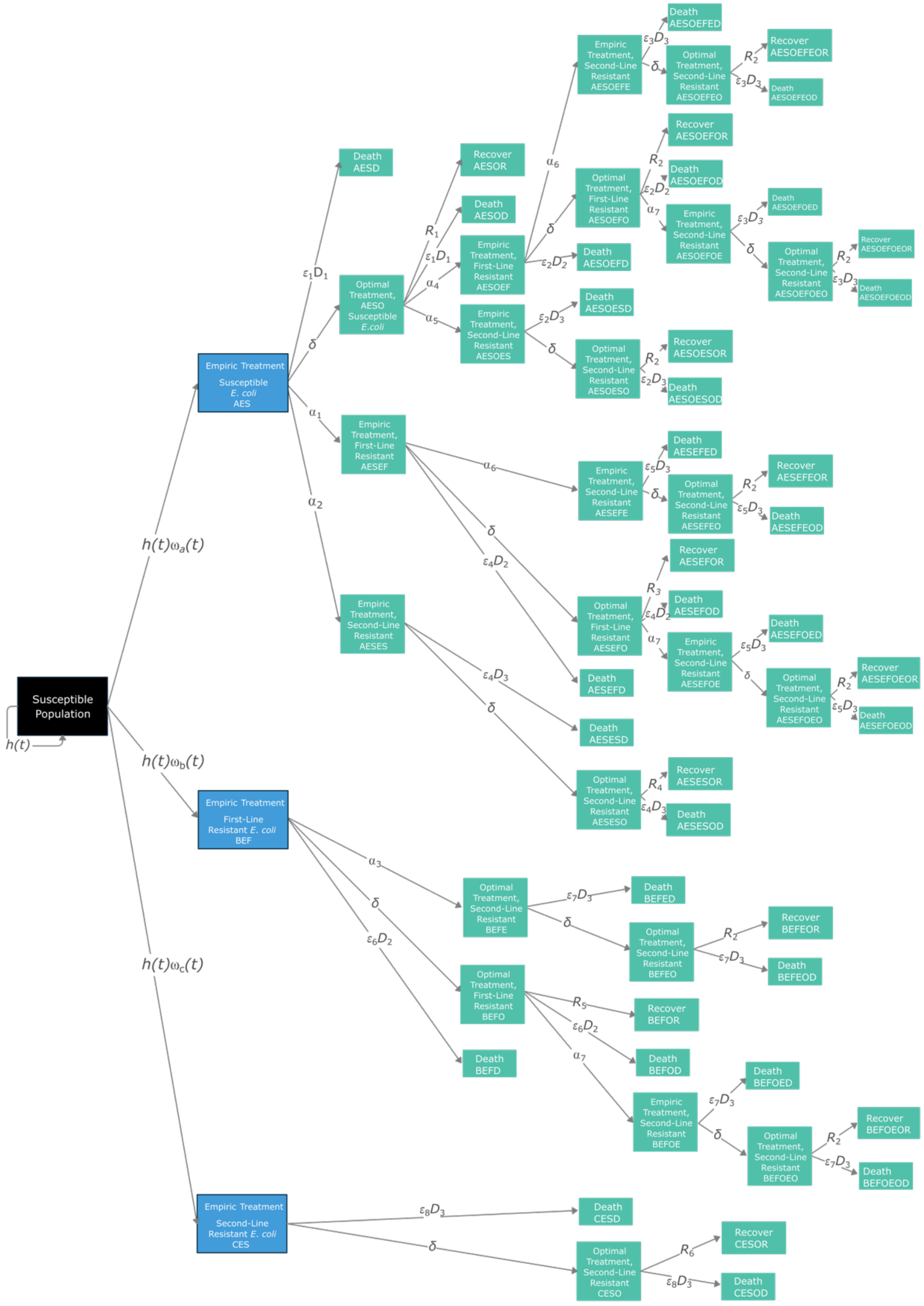

Supplementary S2

Parameter Justification

δ: Rate of switch to optimal treatment: The empiric period for antibiotics is highly variable and context dependent. The empiric period is a function of the time it takes to take a representative sample, for microbiological validation, for resistance testing, for information to be relayed to the clinician, and for optimal treatment to be initiated. Consequently, while growth times for Enterobacteria are around 12 hours,(1) the empiric period often lasts approximately 48 hours,(2) giving δ a value of 0.5. Sensitivity analyses investigate the effect of shorter (i.e., 1 day) and longer (i.e., 4 days) empiric treatment.

ω: Percent of *E. coli* bacteria with certain resistance phenotype: Estimates are sourced from Public Health England's latest ESPAUR laboratory surveillance report.(3) ω_c is the percentage of *E. coli* bacteraemia resistant to second-line therapy, i.e., cefuroxime/gentamicin. According to the report, 5.0% of isolates were resistant to a combination of gentamicin and 3rd generation cephalosporins in 2018, giving ω_c a value of 0.05.[1] 8.6% of isolates were resistant to gentamicin and amoxicillin-clavulanate (first-line therapy), providing a ω_a value of 0.914, or 91.4% (1 - resistance level). While there may be overlaps in resistance profiles, ω_b represents the effective remaining group of patients not falling into the other resistance profiles. This provides a ω_b value of 0.036, or 3.6%. Scenario analyses with different resistance levels for $\omega_{a\text{-}c}$ are explored. Resistance to meropenem (among those resistant to other narrow-spectrum therapies) was <0.1%, and was therefore not included.(3) $\omega(t)$ values are updated over time based on the value of γ:

$$\frac{d\omega_a}{dt} = -\gamma A - \frac{\gamma}{3}B - \frac{\gamma}{3}C$$
$$\frac{d\omega_b}{dt} = \gamma A - \frac{\gamma}{3}B - \frac{2\gamma}{3}C$$
$$\frac{d\omega_c}{dt} = \frac{2\gamma}{3}B + \gamma C$$

Where A, B and C are the number of days treated with first-, second- and third-line antibiotics, respectively, at each time point. γ and the other values in the ODEs are explained in the next section.

[1] Second- and third-generation cephalosporin resistance was assumed to be approximately equal.

γ: **Increase in resistance in population per day of treatment:** Estimating the effect of using antibiotics on the resistance phenotypes of future bacteraemia is difficult. The value of this parameter is akin to a transmission parameter in which antibiotic use leads to increased baseline population-level resistance. The value is difficult to estimate since many factors influence the parameter, including relapses among patients still colonized with *E. coli*, horizontal transfer of plasmids containing ESBL-genes, and other indirect selection pressures.(4–7) Consequently, the value of γ is prone to significant uncertainty.

1.8×10^{-5} was selected as a baseline value. This means that the % resistance towards a given antibiotic increases by 1% per 290,000 doses given. While no exact value exists in the literature, the % increase in resistance per DDD (defined daily dose) per 100 bed days was used to calculate a γ value. An observational study of patients with *E. coli* infections (in Bern, Switzerland, which has a population of roughly the same size of the study population) provided a value of 1.8×10^{-5}.(8) Sensitivity analyses tested values between 1×10^{-5} and 1×10^{-3}.

For the model's equations, the values associated with γ for each antibiotic group (i.e., the relative effects of γ for first-, second- and third-line use) are based on correlation data linking increases in resistance with increasing use. Carbapenems are generally associated with more marked increases in resistance to beta-lactams than cephalosporins and beta-lactam/beta-lactam inhibitors; additionally, the use of aminoglycosides is strongly correlated with reductions in beta-lactamase and ESBL- producing organisms.(9, 10) These dynamics explain the different coefficients in the equations in the previous section (under ω), such as second/third-line use correlated with 1/3 of the increase in first-line resistance compared to first-line use (i.e., using first line antibiotics is associated with a larger increase in first-line resistance compared with second/third-line use).(11, 12) Similarly, second/third-line use is associated with an increase in second-line resistance, whereas first-line use is not. Based on correlation data, third-line (carbapenem) use was assumed to be correlated with twice the increase in resistance to second-line therapy as second-line use.(10) Changes in resistance in the model are relative (i.e., the sum of all groups is equal to 100%).

***h*: Population rate of *E. coli* bacteremia hospitalization:** The aggregate annual rate of *E. coli* bacteraemia for 2022/2023 in the United Kingdom was 68.5 cases per 100,000 people.(13) Converting this to a daily rate (dividing by 365) provides a value of 1.88×10^{-6} *E. coli* bacteraemia cases per person per day in the UK.

λ**: Rate of increase in *E. coli* bacteremia hospitalization rate (per day):** The rate of *E. coli* bacteraemia in the UK has been progressively increasing. The average annual linear increase in rate was calculated between 2021 and 2023, which provided an annual value of 1.7%(13) This was then converted to a daily rate, providing a λ value of 4.66 x 10^{-5}.

***D*: Rate of death per day during treatment:** The baseline death rate for *E. coli* bacteraemia was set at D_1 = 0.01 deaths per day (i.e., 1%) among those with pan-susceptible *E. coli*. This was based on studies of bacteraemia with 7-day mortality rates varying between 6.7% and 8.5%.(14, 15) While most studies state 30-day mortality rates, which vary between 11 and 30.8%, they are more difficult to translate to daily rates since the majority of deaths are skewed towards the first 14 days of illness.(16–19) Baseline death rates for patients with infections resistant to first- and second-line treatment were modelled as higher than the baseline mortality rate, based on evidence of an increased mortality rate among those with bacteraemia infections with beta-lactamase and ESBL-producing organisms.(5, 17, 19, 20) In these studies, the mortality rate ranged from 1.16 to 3.57 times higher among those with beta-lactamase and ESBL-producing organisms. Presumably, this is partially due to poor initial empiric therapy choices - with likely residual confounding. As such, the true effect is likely to be lower than the results above suggest. For this reason, the mortality rates for D_2 (first-line resistant) and D_3 (second-line resistant) were therefore set conservatively at 0.0050 (1.50%/day, 50% higher than D_1) and 0.02 (2%/day, 2x higher than D_1).

ε**: Reduced survival due to inappropriate empiric therapy:** Inappropriate empiric therapy is an independent risk factor for mortality among bacteraemia patients.(21–23) One study found that the 21-day mortality rate was 2.36 times higher among those not receiving adequate antibiotic therapy <72 hours after symptom debut.(24) Two other studies found increases in mortality when appropriate therapy was not initiated within 48 hours at 30% and 40% respectively.(21, 23) Another found much a higher odds ratio estimate at 4.83.(25) Based on this, a conservative estimate of a 30% increase in mortality was set per two days without appropriate therapy. This provides a value for $\varepsilon = 1.3^{(1/\delta)/2}$, where $1/\delta$ is equal to the duration of empiric therapy.

For each of the three scenarios, the values of ε_{1-8} represent the increased risk of mortality for each empiric treatment experienced without optimal therapy. For example, a patient

experiencing no empiric periods without adequate treatment (i.e., the antibiotic covers the resistance phenotype, such as second-line therapy for a pan-susceptible infection), then the value is equal to ε^{o}, or 1. If one empiric treatment is experienced without adequate therapy, then the value is ε^{1} and so on. First-line resistant organisms were assumed to be susceptible to second-line treatment, and second-line resistant infections were assumed to be susceptible to third-line treatment. Different ε values were tested during sensitivity analyses.

***T*: Total treatment duration:** There is considerable debate regarding the optimal treatment duration for *E. coli* bacteraemia, ranging from 5-14 days.(26, 27) The duration of treatment also depends on the clinical status of patients, with recent trials demonstrating that 7 days is noninferior to 14 days for hemodynamically stable patients.(27, 28) A baseline duration was conservatively set at T = 7 days, with 5 and 14 days also tested.

***R*: Rate of recovery:** The rate of recovery is a function of the duration of treatment, T. The value of R is either $1/T$ or $1/(T-(1/\delta))$. The logic behind this is that every patient should ideally complete a full treatment course with an antibiotic that their infection is susceptible to. Additionally, each optimal treatment period is preceded by an empiric period. If the antibiotics received during the empiric period correspond to the optimal treatment (i.e., the organism is susceptible to the empiric treatment), then the optimal treatment period is equal to the total treatment duration minus the duration of the empiric period, since the empiric period would count as part of the treatment duration. This provides an R value of $1/(T-(1/\delta))$. However, if the organism is not susceptible towards the empiric treatment (e.g., a first-line resistant organism being treated with first-line therapy), then the duration of the optimal treatment becomes longer to complete a full course of antibiotic therapy. This results in a value of R equal to $1/T$, where the rate of recovery is lower. The values for R_1 and R_2 are constant in all models. Values for $R_{3\text{-}6}$ are specific to scenarios A to C since the initial empiric treatment differs depending on the scenario.

***α*: Rate of resistance during therapy:** Estimating the rate at which organisms develop resistance is difficult. The rate is dependent on de novo mutations, horizontal transmission of mobile genetic elements (MGE) carrying resistance genes, and resistance fitness costs (i.e., not all mutations are beneficial).(29, 30) It is further complicated by the fact that first- and second-line therapies include two distinct antibiotics; effective resistance thus requires resistance to both compounds. A description of these processes can be found in Supplementary S6.

The largest available review paper (173 studies, 8 antibiotic classes and 225 different treatments) found resistance in 4.0% of organisms and 5.6% of completed treatments, with lower resistance rates found during combination therapy.(31) However, these were not specific to *E. coli* or bacteraemia. In another study of different *Enterobacteriaceae* infections, the overall incidence of resistance was 1.9% during treatment; 5.0% developed resistance to cephalosporins during broad-spectrum cephalosporin treatment while 1.1% developed aminoglycoside-resistance during treatment with aminoglycosides.(32) The median treatment time during the study was 10 days, with no reduction in resistance rates among those receiving combination therapy.(32) Another study looking at *Enterobacter* bacteraemia found that resistance was not less frequent in those receiving combination therapy (cephalosporin and gentamicin) compared to monotherapy.(33) The time to resistance varied between 4 and 18 days, although other studies have found ranges between 1 and 30 days.(33, 34) Based on the evidence above, the lowest resistance rate observed was 1.1% (for aminoglycosides), where the median treatment duration was 10 days in the corresponding study. Therefore, the breakthrough resistance rate during therapy (α) was conservatively set at 0.1%/day (daily rate equal to 1% divided by 10).

The values of $\alpha_{1\text{-}3}$ are unique to each of the scenarios, whereas parameters $\alpha_{4\text{-}7}$ are consistent across all models (Supplementary S4). The different values for $\alpha_{1\text{-}3}$ compared to $\alpha_{4\text{-}7}$ reflect the fact that the selection pressures vary due to the different initial empiric treatments given to patients. An extensive description of the derivations for values $\alpha_{1\text{-}7}$ can be found in Supplementary S5. Different α values were tested during sensitivity analyses.

Supplementary S3

Trends in the rate of *E. coli* bacteraemia in England: 2012/13 to 2022/23

Image taken from Public Health England report(13)

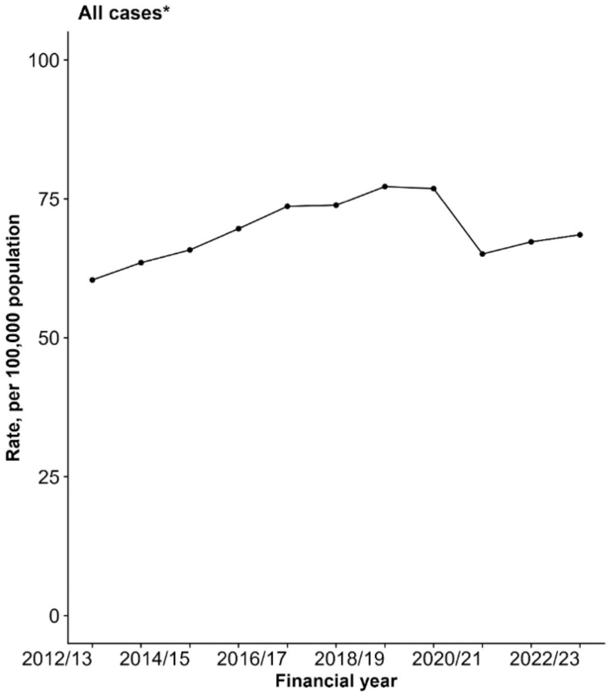

Supplementary S4

Demonstration of which transitions the α values in the model represent.

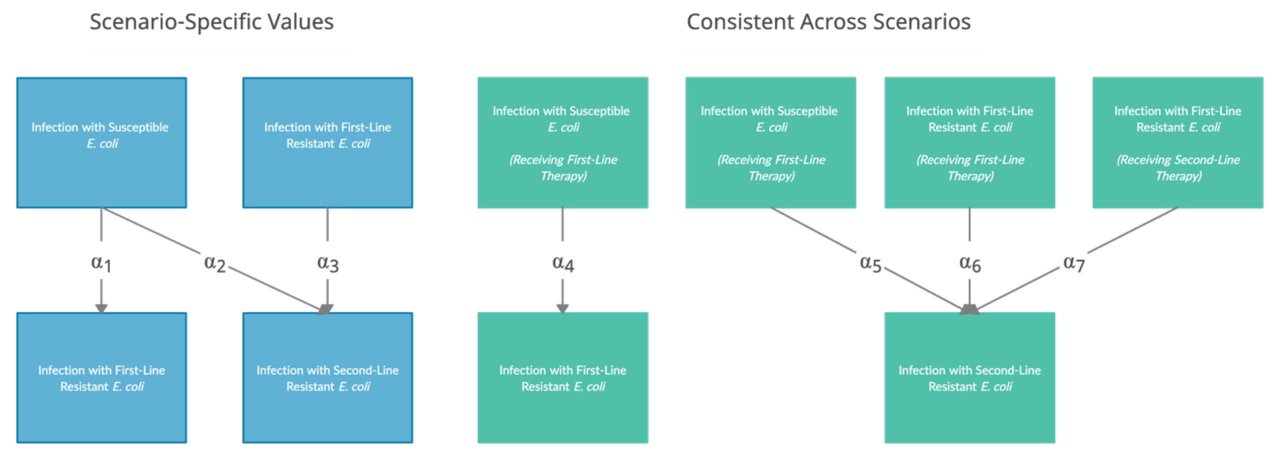

Supplementary S5

Derivations for resistance parameters $\alpha_{1\text{-}7}$

α_4 is the rate at which those receiving optimal treatment for a fully susceptible infection develop resistance to first-line therapy, the value of which is set to the breakthrough resistance rate of α. α_5 is the rate at which those receiving optimal treatment for a fully susceptible infection develop resistance to second-line therapy. Due to limited data for this specific scenario, the likelihood of developing second-line resistance was assumed to be low since the selection pressure for second-line resistance is low. While some resistance may occur mutationally, most is likely to occur through MGEs. The value for α_5 was therefore set conservatively to one-fifth of the breakthrough resistance rate. α_6 represents the rate at which those with infections with first-line resistant microorganisms develop second-line resistance while receiving first-line therapy. Given that the selection pressure is low in this scenario, the value was set equal to α. α_7 represents the rate at which those infected with first-line resistant microorganisms develop second-line resistance while receiving second-line therapy. There is evidence that treating patients with cephalosporins during *E. coli* bacteraemia increases the rate of resistance to cephalosporins by between 2-3 times.(35) However, since the likelihood of resistance here is reduced during combination therapy, the value of α_7 was conservatively set at 50% higher than the value of α.

Baseline values of $\alpha_{1\text{-}3}$ varied between 0 to 50% higher than the breakthrough resistance rate of 0.1%/day. α values 50% higher reflect the same logic as for α_7, whereby the selection pressure increases significantly with broader-spectrum antibiotics. For $\alpha_{1\text{-}2}$ in Scenario B and C, the combined risk was also deemed to be 50% higher. However, since organisms could develop

resistance to either first- or second-line therapy, the risk of developing first-line resistance was deemed to be approximately 3x higher than resistance towards second-line therapy, given the selection pressure and fitness costs. Resistance phenotypes also often emerge through MGEs and are therefore not entirely random unlike mutational resistance. It was also assumed that the likelihood of developing first- or second-line resistance is approximately the same regardless of whether one receives cephalosporin and gentamicin or a carbapenem. This provides α values of 12.5% and 37.5% higher than baseline (total = 50% higher, 37.5% is 3x higher than 12.5%).

References

1. Ruiz-Giardín JM, Martin-Díaz RM, Jaqueti-Aroca J, Garcia-Arata I, San Martín-López JV, Sáiz-Sánchez Buitrago M. 2015. Diagnosis of bacteraemia and growth times. International Journal of Infectious Diseases 41:6–10.

2. Chaubey VP, Pitout JD, Dalton B, Ross T, Church DL, Gregson DB, Laupland KB. 2010. Clinical outcome of empiric antimicrobial therapy of bacteremia due to extended-spectrum beta-lactamase producing Escherichia coli and Klebsiella pneumoniae. BMC Research Notes 3:116.

3. Public Health England. 2019. Laboratory surveillance of Escherichia coli bacteraemia in England, Wales and Northern Ireland: 2018. Health Protection Report, Volume 13, Number 37.

4. Alhashem F, Tiren-Verbeet NL, Alp E, Doganay M. 2017. Treatment of sepsis: What is the antibiotic choice in bacteremia due to carbapenem resistant Enterobacteriaceae ? World Journal of Clinical Cases 5:324.

5. Vihta K-D, Stoesser N, Llewelyn MJ, Quan TP, Davies T, Fawcett NJ, Dunn L, Jeffery K, Butler CC, Hayward G, Andersson M, Morgan M, Oakley S, Mason A, Hopkins S, Wyllie DH, Crook DW, Wilcox MH, Johnson AP, Peto TEA, Walker AS. 2018. Trends over time in Escherichia coli bloodstream infections, urinary tract infections, and antibiotic susceptibilities in Oxfordshire, UK, 1998–2016: a study of electronic health records. The Lancet Infectious Diseases 18:1138–1149.

6. Giannella M, Pascale R, Toschi A, Ferraro G, Graziano E, Furii F, Bartoletti M, Tedeschi S, Ambretti S, Lewis RE, Viale P. 2018. Treatment duration for Escherichia coli bloodstream infection and outcomes: retrospective single-centre study. Clinical Microbiology and Infection 24:1077–1083.

7. Hilty M, Betsch BY, Bögli-Stuber K, Heiniger N, Stadler M, Küffer M, Kronenberg A, Rohrer C, Aebi S, Endimiani A, Droz S, Mühlemann K. 2012. Transmission Dynamics of Extended-Spectrum β-lactamase–Producing Enterobacteriaceae in the Tertiary Care Hospital and the Household Setting. Clinical Infectious Diseases 55:967–975.

8. Cusini A, Herren D, Bütikofer L, Plüss-Suard C, Kronenberg A, Marschall J. 2018. Intra-hospital differences in antibiotic use correlate with antimicrobial resistance rate in Escherichia coli and Klebsiella pneumoniae: a retrospective observational study. Antimicrobial Resistance & Infection Control 7:89.

9. Hsu L-Y, Tan T-Y, Tam VH, Kwa A, Fisher DA, Koh T-H. 2010. Surveillance and Correlation of Antibiotic Prescription and Resistance of Gram-Negative Bacteria in Singaporean Hospitals. Antimicrobial Agents and Chemotherapy 54:1173–1178.

10. Kim B, Kim Y, Hwang H, Kim J, Kim S-W, Bae I-G, Choi WS, Jung SI, Jeong HW, Pai H. 2018. Trends and correlation between antibiotic usage and resistance pattern among hospitalized patients at university hospitals in Korea, 2004 to 2012. Medicine 97:e13719.

11. Wang R, Yang Q, Zhang S, Hong Y, Zhang M, Jiang S. 2019. Trends and correlation of antibiotic susceptibility and antibiotic consumption at a large teaching hospital in China (2007–2016): a surveillance study. Therapeutics and Clinical Risk Management Volume 15:1019–1027.

12. Magee JT, Pritchard EL, Fitzgerald KA, Dunstan FDJ, Howard AJ. 1999. Antibiotic prescribing and antibiotic resistance in community practice: retrospective study, 1996-8. BMJ 319:1239–1240.

13. 2023. Annual epidemiological commentary: Gram-negative, MRSA, MSSA bacteraemia and C. difficile infections, up to and including financial year 2022 to 2023. UK Health Security Agency.

14. Leistner R, Bloch A, Gastmeier P, Schwab F. 2016. E. coli bacteremia in comparison to K. pneumoniae bacteremia: influence of pathogen species and ESBL production on 7-day mortality. Antimicrobial Resistance & Infection Control 5:37.

15. Hattori H, Maeda M, Nagatomo Y, Takuma T, Niki Y, Naito Y, Sasaki T, Ishino K. 2018. Epidemiology and risk factors for mortality in bloodstream infections: A single-center retrospective study in Japan. American Journal of Infection Control 46:e75–e79.

16. Sianipar O, Asmara W, Dwiprahasto I, Mulyono B. 2019. Mortality risk of bloodstream infection caused by either Escherichia coli or Klebsiella pneumoniae producing extended-spectrum β-lactamase: a prospective cohort study. BMC Research Notes 12:719.

17. Melzer M, Petersen I. 2007. Mortality following bacteraemic infection caused by extended spectrum beta-lactamase (ESBL) producing E. coli compared to non-ESBL producing E. coli. Journal of Infection 55:254–259.

18. Laupland KB, Gregson DB, Church DL, Ross T, Pitout JDD. 2008. Incidence, risk factors and outcomes of Escherichia coli bloodstream infections in a large Canadian region. Clinical Microbiology and Infection 14:1041–1047.

19. Abernethy JK, Johnson AP, Guy R, Hinton N, Sheridan EA, Hope RJ. 2015. Thirty day all-cause mortality in patients with Escherichia coli bacteraemia in England. Clinical Microbiology and Infection 21:251.e1-251.e8.

20. Trecarichi EM, Giuliano G, Cattaneo C, Ballanti S, Criscuolo M, Candoni A, Marchesi F, Laurino M, Dargenio M, Fanci R, Cefalo M, Delia M, Spolzino A, Maracci L, Nadali G, Busca A, Del Principe MI, Daffini R, Simonetti E, Dragonetti G, Zannier ME, Pagano L, Tumbarello M. 2019. Bloodstream infections caused by Escherichia coli in onco-

haematological patients: Risk factors and mortality in an Italian prospective survey. PLOS ONE 14:e0224465.

21. Chen F-C, Ho Y-N, Cheng H-H, Wu C-H, Change M-W, Su C-M. 2020. Does inappropriate initial antibiotic therapy affect in-hospital mortality of patients in the emergency department with Escherichia coli and Klebsiella pneumoniae bloodstream infections? International Journal of Immunopathology and Pharmacology 34:205873842094237.

22. Tacconelli E, Cataldo MA, Mutters NT, Carrara E, Bartoloni A, Raglio A, Cauda R, Mantengoli E, Luzzaro F, Pan A, Beccara LA, Pecile P, Tinelli M, Rossolini GM. 2019. Role of place of acquisition and inappropriate empirical antibiotic therapy on the outcome of extended-spectrum β-lactamase-producing Enterobacteriaceae infections. International Journal of Antimicrobial Agents 54:49–54.

23. Kang C-I, Kim S-H, Park WB, Lee K-D, Kim H-B, Kim E-C, Oh M, Choe K-W. 2005. Bloodstream Infections Caused by Antibiotic-Resistant Gram-Negative Bacilli: Risk Factors for Mortality and Impact of Inappropriate Initial Antimicrobial Therapy on Outcome. Antimicrobial Agents and Chemotherapy 49:760–766.

24. De Rosa FG, Pagani N, Fossati L, Raviolo S, Cometto C, Cavallerio P, Parlato C, Guglielmi E, Serra R, Di Perri G. 2011. The effect of inappropriate therapy on bacteremia by ESBL-producing bacteria. Infection 39:555–561.

25. Ortega M, Marco F, Soriano A, Almela M, Martínez JA, Muñoz A, Mensa J. 2009. Analysis of 4758 Escherichia coli bacteraemia episodes: predictive factors for isolation of an antibiotic-resistant strain and their impact on the outcome. Journal of Antimicrobial Chemotherapy 63:568–574.

26. Riordan A. 2016. 5, 7, 10 or 14 days: appropriate duration of treatment for bacteraemia or an example of ‘antimicrobial bingo’? Archives of Disease in Childhood 101:117–118.

27. Yahav D, Franceschini E, Koppel F, Turjeman A, Babich T, Bitterman R, Neuberger A, Ghanem-Zoubi N, Santoro A, Eliakim-Raz N, Pertzov B, Steinmetz T, Stern A, Dickstein Y, Maroun E, Zayyad H, Bishara J, Alon D, Edel Y, Goldberg E, Venturelli C, Mussini C, Leibovici L, Paul M. 2019. Seven Versus 14 Days of Antibiotic Therapy for Uncomplicated Gram-negative Bacteremia: A Noninferiority Randomized Controlled Trial. Clinical Infectious Diseases 69:1091–1098.

28. Havey TC, Fowler RA, Daneman N. 2011. Duration of antibiotic therapy for bacteremia: a systematic review and meta-analysis. Critical Care 15:R267.

29. Hoeksema M, Jonker MJ, Bel K, Brul S, ter Kuile BH. 2018. Genome rearrangements in Escherichia coli during de novo acquisition of resistance to a single antibiotic or two antibiotics successively. BMC Genomics 19:973.

30. Bleibtreu A, Gros P-A, Laouénan C, Clermont O, Le Nagard H, Picard B, Tenaillon O, Denamur E. 2013. Fitness, Stress Resistance, and Extraintestinal Virulence in Escherichia coli. Infection and Immunity 81:2733–2742.

31. Fish DN, Piscitelli SC, Danziger LH. Development of resistance during antimicrobial therapy: a review of antibiotic classes and patient characteristics in 173 studies. Pharmacotherapy 15:279–91.

32. Choi S-H, Lee JE, Park SJ, Choi S-H, Lee S-O, Jeong J-Y, Kim M-N, Woo JH, Kim YS. 2008. Emergence of Antibiotic Resistance during Therapy for Infections Caused by Enterobacteriaceae Producing AmpC β-Lactamase: Implications for Antibiotic Use. Antimicrobial Agents and Chemotherapy 52:995–1000.

33. Chow JW. 1991. Enterobacter Bacteremia: Clinical Features and Emergence of Antibiotic Resistance during Therapy. Annals of Internal Medicine 115:585.

34. Chotiprasitsakul D, Han JH, Cosgrove SE, Harris AD, Lautenbach E, Conley AT, Tolomeo P, Wise J, Tamma PD, Antibacterial Resistance Leadership Group. 2018. Comparing the Outcomes of Adults With Enterobacteriaceae Bacteremia Receiving Short-Course Versus Prolonged-Course Antibiotic Therapy in a Multicenter, Propensity Score–Matched Cohort. Clinical Infectious Diseases 66:172–177.

35. Martinez JA, Aguilar J, Almela M, Marco F, Soriano A, Lopez F, Balasso V, Pozo L, Mensa J. 2006. Prior use of carbapenems may be a significant risk factor for extended-spectrum -lactamase-producing Escherichia coli or Klebsiella spp. in patients with bacteraemia. Journal of Antimicrobial Chemotherapy 58:1082–1085.
